# How do breast cancer surgery scars impact survivorship? Findings from a nationwide survey in the United States

**DOI:** 10.1186/s12885-019-5553-0

**Published:** 2019-04-11

**Authors:** Jennifer Gass, Sunny Mitchell, Michael Hanna

**Affiliations:** 1 Department of Obstetrics and Gynecology, Woman & Infants Hospital, Women & Infants Breast Health Center, 1 Blackstone Place, 2nd floor, Providence, RI 02905 USA; 2 Department of Surgery, Alpert Medical School, Brown University, Providence, RI USA; 3 Independent Researcher, Stratford, CT USA; 4 Mercury Medical Research & Writing, New York, NY USA

**Keywords:** Breast cancer, Surgery, Mastectomy, Lumpectomy, Patient survey, Scars, Survivorship

## Abstract

**Background:**

The surgical treatment of breast cancer has been associated with negative consequences for patients’ body image, sexual functioning, mental health, and social adjustment. Recent advances in the surgical approach to breast cancer allow the oncologic surgeon to safely optimize cosmetic outcomes. Little is known about the possible relevance of surgical scars. The aim of this research was to gather the perspective of breast cancer survivors themselves on the issue of surgical scars and their negative impact on survivorship.

**Methods:**

An internet survey was conducted nationwide in the United States among women who reported being surgically treated by lumpectomy, mastectomy, or both procedures for breast cancer. To improve generalizability, census-based enrollment quotas were applied for geographic region, health insurance, and income.

**Results:**

The five hundred respondents reported lumpectomy only (*n* = 215), mastectomy only (*n* = 140), or both surgeries (*n* = 132). In response to the statement, “I do not like the location of my surgical scar”, 64% of lumpectomy-only respondents and 67% of mastectomy-only respondents agreed somewhat or strongly. Only 26% of lumpectomy respondents and 14% of mastectomy respondents reported minimal or no negative impact as a consequence of the surgical scars.

**Conclusion:**

Consistent with previous literature, this nationwide US survey shows that the majority of women feel negatively affected by their breast cancer surgery scars. Surgeons should consider this outcome when planning surgery, which may improve patients’ survivorship journey.

**Electronic supplementary material:**

The online version of this article (10.1186/s12885-019-5553-0) contains supplementary material, which is available to authorized users.

## Background

Breast cancer is the second most common type of cancer, with 230,815 newly diagnosed cases in women in the USA in 2013 [1, 2]. Most new cases are early-stage tumors amenable to successful treatment with excellent long-term survival [3–5]. Surgical removal of the tumor is a key step of treatment. [6–9] Advances in surgical treatment have progressively reduced morbidity over the years while maintaining or improving survival rates [10–15]. Breast-conserving surgery, nipple-sparing mastectomy, and sentinel-node biopsy have become established approaches that have reduced surgical morbidity. Since survival rates today are already quite high (98.9% for stage 1 breast cancer and 85.2% for stage 2 at 5 years [16]), recent advances in breast cancer surgery techniques have focused increasingly on reducing surgical morbidity and improving survivors’ quality-of-life.

Despite substantial reductions of surgical morbidity in our era, studies suggest an association between surgery and negative psychosocial effects for many breast cancer patients, particularly in regards to body image [17–22], sexual relations [17, 18], and mental health [17, 19]. Two methodologically strong, population-based, 5-year longitudinal studies in Germany both consistently found that mastectomy had more impact than breast-conserving surgery on several domains, including body image, role functioning, and sexual activity, among others. [23, 24] Both studies also indicated (albeit with less consistency in the details) that patients who had breast-conserving surgery were more likely to show improvement over time. The better psychosocial outcomes for breast-conserving therapy compared to mastectomy do indeed suggest, in a general way, that the degree of psychosocial impact is related to the physical severity of the surgery, and not merely to the overall experience of cancer or to pre-diagnosis patient factors. But it remains unclear which specific physical aspects have the most influence on which specific psychosocial outcomes. A literature review concluded that most of the studies of the effects of breast cancer surgery on quality of life have been “inadequate for various [methodological] reasons” and “better delineation of surgical outcomes and their effects is needed to achieve improved quality of life” [25].

And unfortunately those previous studies did not assess the impact of surgical scars specifically. Two small qualitative studies have provided many insights about the relevance of scarring for breast cancer survivors [26, 27], but it is very unclear if their findings would be broadly generalizable to other patients. A recent clinical study in Sweden found that 81% of 297 women who underwent breast-conserving surgery (at a median age of 62) were satisfied or very satisfied with the appearance of their surgical scar at a median follow-up time of 16 months post-op (while another 8% were missing data) [28]. They also found that an estimated percentage of breast volume excised ≥20% was a risk factor for dissatisfaction with the scar and with overall aesthetic outcome. A survey in Ireland of 312 women without a history of breast cancer, 88 women with a history of breast cancer, and 100 male partners examined the respondents’ preferences about scarring for hypothesized surgical treatment of breast cancer [29]. They found that 66% of the women thought postsurgical scars would not be important, (while 73% of men thought they would be). But since only 22% of the women had actually had a history of breast cancer, that result only gives an indication of how many women think scarring would be important, not how often scarring actually does have an impact. The survey also found that the majority of respondents would prefer the scar in the lower lateral quadrant of the breast, and would prefer a circumareolar scar, with nearly identical results for women and men on these two points. The authors interpreted these findings as indicating a preference for scars with less visibility. They concluded that there is a dearth of information on scarring and further research is needed on patients’ feelings about scars.

The aim of the nationwide survey research reported in this paper was to explore how women themselves feel specifically about the scars from their breast cancer surgery and if/how those scars might affect their lives. In other words: “Do scars matter?”

## Methods

### Ethics

Because this research only involved anonymous public surveying, it was exempt from ethics committee approval, according to the Code of Federal Regulations, Title 45 – Public Welfare Department of Health and Human Service, Part 46 – Protection of Human Subjects, section 46.101.b.2 [30].

### Study design

The study was designed as a survey, in order to obtain quantitative results – from the patient perspective – that would be generalizable to the larger population. The survey was conducted by internet, nationwide in the United States, 13–24 June 2016, by a well-established public surveying company.

### Participants

The pool of potential participants for this survey had previously agreed to be contacted about participation in public surveys. Participation in this survey was anonymous and voluntary. Consent to participate in this specific survey was implicit in each participant’s choice to continue the survey beyond the initial welcome page explaining it. Further information about participant recruiting and consent has been reported previously [31].

Respondents were eligible to participate if they reported: 1) female gender, 2) an age of 18–99, 3) ever having been diagnosed with breast cancer, and 4) having undergone lumpectomy or mastectomy. Enrollment quotas were set for health insurance status, income level, and the four geographic regions (Northeast, South, Midwest, West), using information from the US government Census Bureau, so that the originally intended total sample (N = 500) would reflect those demographics of the general US population. When quotas were reached for subgroups on those parameters, the participation of further subjects who were “over quota” was terminated, and their survey data were not used.

Enrollment limits were also set at 250 participants for the lumpectomy survey and 250 for the mastectomy survey. If a participant reported both lumpectomy and mastectomy, she was assigned to the mastectomy survey until that survey reached its enrollment limit; thereafter, such women were assigned to the lumpectomy survey. The original rationale for the sample size was explained previously [31].

### Questionnaire

The questionnaire is presented in Additional file 1. It had three sections. The first section contained 7 screening and demographic questions. Participants were then instructed to think of their most recent lumpectomy / mastectomy, depending on their survey assignment. The second section contained 16 content questions, but participants were asked only 13 or 14 of those questions, depending on their answers. This paper reports only content questions 8–16; (the results of content questions 1–7 have been reported previously [31]). The third section contained 9 demographic questions. Information about the development of the survey questions has been reported previously [31].

### Statistical analysis

Descriptive statistics were used to characterize the study sample and summarize their responses. In order to assess the precision of our survey results, 95% confidence intervals (95% CIs) were calculated for the descriptive results of some key outcomes, using bootstrapping with 1000 iterations of the sample [32, 33]. Multivariable logistic regression analysis was performed to determine if the response to the main outcome about the location of the scar (Q12) depended on various other demographic predictor variables. The number of predictor variables tested in the model depended on the descriptive results to the outcome variable, according to the generally accepted guideline of 10 events per variable [34–39]. Also as per best practices, the predictor variables were selected for testing in the regression model based on our expectations of their relevancy, not on prior statistical testing [37–40].

### Reporting

The study has been reported according to recommended best practices and proto-guidelines in the literature for the reporting of survey research [41–44]. A previous paper has reported further details about the methods and study sample, as well as the survey results about pre-operative informing [31].

## Results

### Study enrollment

The survey was completed by 215 women who reported undergoing lumpectomy only, 140 women who reported mastectomy only, and 132 women who reported both surgeries. Further information about the flow of participants from recruitment to survey completion, including statistical calculations and explanations about that, has been reported in detail previously [31]. Given the difficulty in determining which surgery the “both surgeries” respondents were thinking about, as confirmed by three peer reviewers, we decided to present the results and discussion of these 132 women who had both surgeries in additional file 2. For reading convenience, we present the results of the two main study groups side-by-side, but we advise against comparing the two groups to each other, mainly because this study is not a clinical trial comparing lumpectomy to mastectomy and that is not the point of the analysis. Instead, we encourage readers to assess the results from each of the study groups independently, relative to current consensus of what high-quality cancer care should achieve.

### Study sample characteristics

The demographic and healthcare characteristics of the study sample are presented in Table 1.

**Table 1:** Demographic and Healthcare Characteristics of the Study Sample. In the rows, the demographic characteristics are presented in bold, subtotals of various answer options (if any) are presented in italics, and original answer options are presented in smaller roman type. The results presented are the percentage of the total for that study group (lumpectomy only n = 215, mastectomy only n = 140). For the sake of reading simplicity, we do not also present the actual number of subjects, but they are available upon request. Due to rounding off, the percentages may not always add up exactly to 100% (or other indicated subtotals)

	Lumpectomy only	Mastectomy only
Age		
*18–29*	*9*	*11*
*30–39*	*29*	*21*
*40–49*	*31*	*25*
*50–59*	*8*	*21*
*60–69*	*12*	*16*
*70–89*	*10*	*6*
Race		
*Majority Subtotal*	*85*	*81*
White or Caucasian	85	81
*Minority Subtotal*	*15*	*19*
African American	10	13
Asian	1	4
Native American	0	2
Other	3	0
Ethnicity		
Not Hispanic, Latino, or Spanish Descent	82	91
Hispanic, Latino, or Spanish Descent	18	9
Education		
*College Graduate Subtotal*	*69*	*56*
Graduate or post-graduate work	16	10
Graduated from college	53	46
*Not College Graduate Subtotal*	*31*	*44*
Some College	16	20
Technical or vocational school	2	3
Graduated from high school	11	20
Some high school	1	1
Grade school	0	0
Employment		
*Working Full-Time Subtotal*	*67*	*61*
Work full-time	67	61
*Not Working Full-Time Subtotal*	*33*	*39*
Work part-time	7	9
Unemployed	5	2
Retired	14	19
Stay-at-home / do not work	7	9
Income (total household income for last year) ^a^		
Less than $35,000	13	13
$35,000 – $49,999	20	19
$50,000 – $74,999	19	30
$75,000 – $99,999	15	25
$100,000 – $149,999	18	11
$150,000 or more	15	2
Residential Area Type		
City / urban area	54	39
Suburbs	39	44
Rural area (e.g. very small town or farm)	7	17
Marital Status		
*Has Significant Other Subtotal*	*74*	*58*
Married or living as married	69	55
In a relationship	5	3
*Does Not Have Significant Other Subtotal*	*26*	*42*
Single	14	28
Separated	1	2
Divorced	4	7
Widowed	7	5
Parental Status		
No children under 18 living in her home	45	66
Has child under 18 living in her home	55	34
Covered by Health Insurance or Health Care Plan		
Yes	95	98
No	5	2
Breast Cancer Treatments Undergone		
Chemotherapy	38	51
Radiation therapy	46	37
Hormone therapy	19	19
Targeted therapy	12	9
Bone-directed therapy	1	1

a) To put the income results into context: the US government reports that the median household income in 2015 was $55,775, and the weighted average poverty threshold was $12,082 for a one-person household, $15,391 for a two-person household, $24,036 for a four-person household in which two were children under 18, and $35,473 for a seven-person household in which five were children under 18

https://www.census.gov/content/dam/Census/library/publications/2016/acs/acsbr15-02.pdf

http://www.census.gov/data/tables/time-series/demo/income-poverty/historical-poverty-thresholds.html

### Survey responses: psychosocial impact of surgical scars

**Fig. 1:**
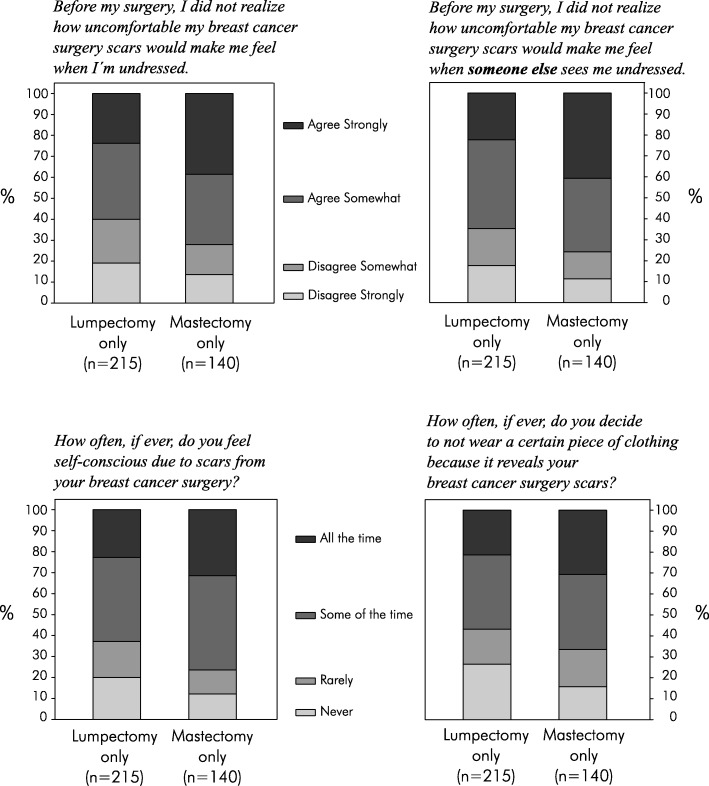
Stacked horizontal bar charts for the results to Q15 (top left), Q16 (top right), Q13 (bottom left), and Q14 (bottom right). Note 1: For Q15, the bootstrappped 95%CI for the answer “agree strongly” was 18–29% for lumpectomy-only patients and 31–46% for mastectomy-only patients. For Q16, the bootstrappped 95%CI for the answer “agree strongly” was 17–28% for lumpectomy-only patients and 32–49% for mastectomy-only patients. For Q13, the bootstrappped 95%CI for the answer “all the time” was 18–28% for lumpectomy-only patients and 24–39% for mastectomy-only patients. For Q14, the bootstrappped 95%CI for the answer “all the time” was 16–27% for lumpectomy-only patients and 23–39% for mastectomy-only patients. Note 2: The two survey questions in the top row may seem to be very similar, and the overall distribution of answers may also seem very similar. But these two survey items addressed different aspects (body image vs. intimacy), and a not-negligible portion of survey participants gave different answers on these two items. Among the lumpectomy-only patients, 135 (63%) gave the same answer to both statements; 42 (20%) either agreed to both or disagreed to both but with different strengths; and 38 (18%) agreed with one statement and disagreed with the other. Among the mastectomy-only patients, 99 (71%) gave the same answer to both statements; 24 (17%) either agreed to both or disagreed to both but with different strengths; and 17 (12%) agreed with one statement and disagreed with the other

The majority of both study groups agreed that before surgery they did not realize how uncomfortable their breast cancer surgery scars would make them feel when undressed and also when someone else sees them undressed (Fig. 1**,** upper row). The majority of both study groups felt self-conscious due to scars from their breast cancer surgery some or all of the time, and they also decided some or all of the time to not wear certain pieces of clothing because it reveals their breast cancer surgery scars (Fig. 1**,** lower row). The portion of women who disagreed somewhat or strongly to both questions in the upper row of Fig. 1 and also responded “rarely” or “never” to both questions in the lower row of Fig. 1 was 25.6% in the lumpectomy-only group and 14.3% in the mastectomy-only group. These are the rates of breast cancer survivors for whom surgical scars did not have any clinically meaningful impact, as far as our survey was able to assess.

### Survey responses: feelings about the scar location

The majority of women agreed with the statement, “I do not like the location of my surgical scar” (Q12) (Table 2). Demographically, some types of women were more likely than others to agree strongly with that statement (Table 3).

**Table 2:** Descriptive Results for Agreement / Disagreement with the Main Outcome, “I do not like the location of my surgical scar” (Q12). This table presents the basic descriptive results to this survey question. In the left-hand column, study groups are written in bold; beneath them are the answer options of the survey. The further columns present the n, the %, and the bootstrapped 95% Confidence Interval of the % of respondents. [Recall: in simple terms, the 95% CI represents how much the results might have been different in other hypothetical study samples. If we were to repeat this survey 1000 times in different study samples drawn from the same population, then the results of 950 of those surveys would be somewhere within the 95% CI. The other 50 surveys would be outside the 95% CI (25 would be even lower and the other 25 would be even higher). So for example: in our survey, 20% of the lumpectomy-only patients agreed strongly. If we repeated this survey 1000 times with different women drawn from the same population, we would expect that the results from 950 of those 1000 surveys would be somewhere between 15 and 25% of the lumpectomy-only patients agreeing strongly, as shown in the table. Twenty-five surveys would be even less than 15%, and twenty-five surveys would be even higher than 25%. Expressed another way, we might say that this specific result – that 20% of lumpectomy-only patients agreed strongly – has a margin of error of ±5% of the respondents.]

	n	%	95% CI
Lumpectomy Only (*n* = 215)			
Agree strongly	43	20	15–25
Agree somewhat	94	44	38–50
Disagree somewhat	46	21	16–27
Disagree strongly	32	15	11–21
Mastectomy Only (*n* = 140)			
Agree strongly	46	33	24–41
Agree somewhat	47	34	26–42
Disagree somewhat	33	24	16–31
Disagree strongly	14	10	5–15

**Table 3:** Results of the regression analysis for strong agreement with the statement, “I do not like the location of my surgical scar” (Q12). In the left-hand column, study groups are written in bold; beneath them are all the independent predictor variables tested in the model (in order of decreasing significance). Predictor variables in gray were clearly not statistically significant (p ≥ 0.1). The further columns present the odds ratio, the 95%CI of the OR, and the p-value. [Recall the following about odds ratios: a) an OR of 1.0 would mean that the predictor variable had no effect on the outcome variable; b) an OR of 2.0 would mean that if the predictor variable was present (or for each unit of increase for ordinal or continuous variables, e.g. each year for age), then there were double the odds for the outcome variable of strongly agreeing with the statement of not liking the location of the scar; c) an OR of 0.5 would mean that if the predictor variable was present (or for each unit of increase for ordinal or continuous variables, e.g. each year for age), then there were half the odds of the outcome variable of strongly agreeing with the statement of not liking the location of the scar. So for example, among the patients who had mastectomy only, college graduates had 0.3 times lower odds of strongly agreeing with the statement, “I do not like the location of my scar.” It is important to keep in mind that the OR for a continuous variable, such as age, is for each increase of one unit (here, a year of age) and cumulative. So for example, among lumpectomy-only patients, women who were 10 years older (than another woman of whatever age) would have an OR of (0.96)^10^ = 0.66, and women who were 25 years older would have an OR of (0.96)^25^ = 0.36; among mastectomy-only patients, women who were 10 years older would have an OR of (0.97)^10^ = 0.72, and women who were 25 years older would have an OR of (0.97)^25^ = 0.45.]

	OR	lower 95%CI	upper 95%CI	p
Lumpectomy Only (*n* = 215)				
Age (years)	0.96	0.94	0.99	0.010
Significant Other	2.3	0.86	6.3	0.098
Income (6 brackets)	0.9	0.7	1.2	0.5
College Graduate	0.7	0.3	1.8	0.5
Mastectomy Only (*n* = 140)				
College Graduate	0.3	0.1	0.7	0.006
Age (years)	0.97	0.94	0.999	0.04
Income (6 brackets)	0.8	0.6	1.2	0.4
Significant Other	1.2	0.6	2.7	0.6

Note: The regression model fit the lumpectomy data well according to Pearson chi-Square (203, p = 0.6), likelihood ratio (10.9, p = 0.03), and Hosmer-Lemeshow (6.5, p = 0.6). The regression model fit the mastectomy data well according to Pearson chi-Square (138, p = 0.4) and likelihood ratio (14.7, p = 0.005) but was perhaps a marginally poor fit according to Hosmer-Lemeshow (15.2, p = 0.056)

### Survey responses: informing about surgical options

**Fig. 2:**
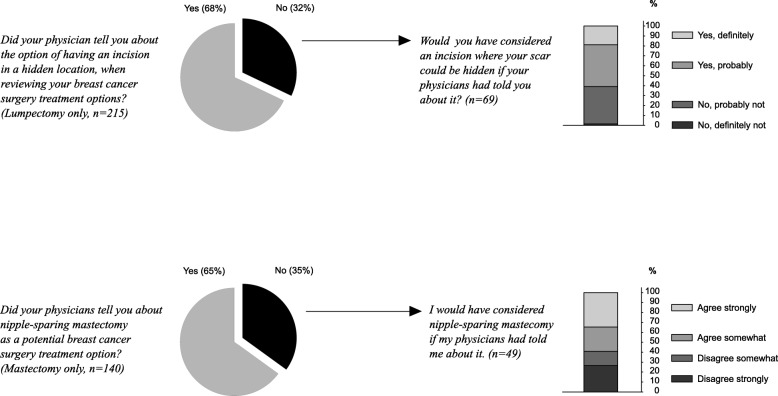
Response to the Survey Question on Informing about Surgical Options. The top row presents the results for patients who reported lumpectomy only; the bottom row presents results for patients who reported mastectomy only. The left side presents pie charts of how many women reported that their physician told them about the surgical option in question. Patients who reported that they were not told about that surgical option were then asked if they would have considered it, if they had been told. The right side presents a stacked bar chart of the answers of those subsamples. It should be kept in mind that some of the women who did not hear about these options may simply have not been indicated for them, or may have had their surgery before the option became widely available

Approximately one-third of the lumpectomy-only patients said that they were not informed about the option of having the surgical incision in a hidden location, and about one-third of mastectomy patients said they were not informed about nipple-sparing mastectomy (Fig. 2**,** left side). It is possible that some portion of these patients were not informed of these options because they were not eligible for them. Among these subgroups who said that they were not told about these options, the majority said that they would have considered these options if they had been told (Fig. 2, right side).

## Discussion

The goal of breast cancer surgery is complete tumor excision. Because the five-year survival rates are already quite high (98.9% for stage 1 and 85.2% for stage 2 [16]), current-era surgery strives to minimize morbidity. The Institute of Medicine mandates that healthcare providers consider the long-term side-effects of cancer therapy. [45]. Recent guidelines for survivorship from the National Comprehensive Cancer Network also emphasize the important of addressing the late consequences of cancer treatment [46]. One of these long-term side-effects might be the possible psychosocial impact of the surgical scarring. Minimizing surgical morbidity by maintaining the patient’s natural breast began with breast-conserving surgery and progressed further with skin-sparing and then nipple-sparing mastectomy. Turning to breast-conserving surgery specifically, emphasis on the aesthetic outcomes of the breast has lagged behind the advances seen in mastectomy.

Historically, the scarring of breast cancer surgery has been viewed by the surgical community as predominantly a cosmetic consequence [47–54]. The primary metric was “how the woman’s chest appears visually in the external world, dressed and covered” – including to the patient herself. In today’s world, how a breast appears dressed/covered may not be the only or most relevant endpoint. Therefore, rather than external evaluation of the patients’ physical appearance, this survey assessed these surgical scars from the perspective of the patient herself, in regards to her own lived daily experiences. Consistent with an emerging literature, [23, 26–29] our study found indications that surgical scaring is not merely an inconsequential issue of “cosmetic” or “aesthetic” appearance, as one might at first assume. The majority of women in this study reported that their breast cancer surgery scars made them feel uncomfortable when they are undressed and when someone else sees them undressed (Fig. 1, upper row) – thus impacts in the domains of body image and intimacy, respectively. The majority of women in this study also reported that their scars made them feel self-conscious and avoid certain pieces of clothing, some or all of the time (Fig. 1, lower row) – thus impacts in the domains of psychological state and social presentation, respectively. Only a small minority of women were able to avoid all four of these impacts from scarring.

In both survey groups, the majority of women agreed that they did not like the location of the scar. The reliability of this result is demonstrated by the narrow 95% confidence intervals reported and explained in Table 2. Due to our survey sample size, we were only able to test a small number of possible predictor factors in the regression analysis. Nonetheless, we found that older women were less likely to agree strongly that they did not like the location of the scar, and the odds were nearly cut in half (cumulative OR ≈ 0.5) for age differences of about 17 years for lumpectomy only and about 23 years for mastectomy only. (For example, a 57 year old woman with lumpectomy only would have about half the odds of agreeing strongly with that statement, compared to a 40 year old woman with lumpectomy only.) So overall, the majority of women agreed that they did not like the location of the scar, and strong agreement was more frequent among younger women.

Historically, the standard surgical technique placed the incision directly anterior to the tumor. Today, oncoplastic surgery, with tissue mobilization and scars unaligned to the tumor bed, has gained acceptance, with a recent metaanalysis of several studies documenting its safety [5]. Therefore, given that scar placement does not need to be anterior to the tumor, patients can be offered lumpectomy through a less visible scar that reduces the surgical imprint. Anatomically ideal locations include the axillary fossa, periareola border, and inframammary fold. Current surgical techniques and operating room technologies enable surgeons often to perform complete tumor excision with clear margins through remotely placed incision(s) [55]. Whether the procedure is lumpectomy or mastectomy, placement of the incision in an anatomically ideal location might reduce the consequences of surgical scars, while still accomplishing the goal of complete tumor excision. But our survey suggests that many women may not have been informed about the options of hidden-incision lumpectomy or nipple-sparing mastectomy (Fig. 2), consistent with the broader deficits in pre-operative informing that we reported previously [31].

The study limitations and strengths have been discussed previously [31]. Briefly, the main limitations are that this survey: 1) was cross-sectional not longitudinal, 2) was conducted at one brief time without any relation to the respondents’ surgical dates, 3) may not be generalizable to many kinds of breast cancer patients who demographically could not possibly have participated in this survey, 4) may have had a selection bias against women in poorer health, 5) used an unvalidated questionnaire, and 6) relied upon self-reported medical information. Additionally for this paper: 7) the specific surgical techniques actually used, (including oncoplasty, nipple-sparing mastectomy, reconstruction, etc.) remain unknown; 8) this survey did not assess objective physical characteristics of the patients’ scars [56], functional impairment, pain, or other physical sensations [27] for further analysis in relation to the reported subjective impact of the scars; and 9) this study was not designed to assess their preoperative body image and psychosocial state, which may also have influenced their reactions to the surgical scar. The study strengths, again only briefly summarizing, are that this survey: 1) provides the valuable patient perspective, 2) was nationwide in the United States and representative for many demographic variables, 3) was administered by an established surveying firm using a carefully worded questionnaire, 4) achieved sufficiently high rates of participation and completion, 5) had no missing data, and 6) yielded 95% CIs with sufficient narrowness that the conclusions would not change anywhere within those ranges. A previous report provides fuller explanations about these methodological aspects [31].

This study provides initial indications that surgical scars can have a negative impact on breast cancer survivors. Further research would advance our understanding of this topic. Clinical research focused specifically on the impact of scars should assess various components of both the preoperative psychosocial state of the patient and the objective physical characteristics of the scar, and then attempt to disentangle their relative contributions to the postoperative psychosocial effects of the scar on the patient. Clinical trials would also be an important step toward quantifying the magnitude and nature of the possible advantages of placing surgical incisions in better anatomical locations. Multidisciplinary studies could assess the possible benefits of supplemental psychosocial counseling focused specifically on adapting to the surgical process and its effects. A small minority of women reported minimal or no psychosocial impact of scars; more in-depth research on such women might yield valuable insights about resilience that could improve the psychosocial care of other patients.

## Conclusions

This portion of our survey explored the research question, “Do surgical scars matter to breast cancer survivors?” For the majority of respondents (74% of lumpectomy only patients and 86% of mastectomy patients), “Yes, scars do matter”, and surgical scars are associated with a negative impact in their daily lives. Our work introduces the concept that breast cancer surgery scars may be thought of as morbidity for breast cancer patients. Pre-operative informed consent is the ideal opportunity to address this concern. Finally, surgeons have an opportunity to reduce this morbidity by placing the surgical incision in a less visible location, and this may be one additional way that surgeons can improve survivorship outcomes for many women.

## Additional files

Additional file 1: Questionnaire. This file provides the entire survey questionnaire that was used in this study. (PDF 73 kb)

Additional file 2: Women Reporting Both Surgeries. This file provides background, results, and commentary on the 132 women who reported both lumpectomy and mastectomy. (PDF 238 kb)

## Abbreviations

CI: Confidence Interval
n: number
OR: Odds Ratio
