# Supplementary material for: How do breast cancer surgery scars impact survivorship? Findings from a nationwide survey in the United States
Source: BMC Cancer. 2019 Apr 11;19:342. doi: 10.1186/s12885-019-5553-0 (PMC6458748; doi:10.1186/s12885-019-5553-0)
Supplement: Supplementary file 1 — Questionnaire. This file provides the entire survey questionnaire that was used in this study. (PDF 73 kb) [file 12885_2019_5553_MOESM1_ESM.pdf]

Supplemental File 1: Questionnaire.

Note: Neither the text written in all capital letters nor the numbers of the response options were shown to the survey participants.

SCREENER QUESTIONS

GENDER. What is your gender please?

1) Male TERMINATE
2) Female

AGE. What is your age? OPEN END NUMERIC, MIN=18, MAX=99

STATE. What state do you live in currently? 50 STATES + DC

A. Which of the following conditions, if any, have you ever been diagnosed with? Please select all that apply. RANDOMIZE, MULTI RESPONSE, TERMINATE IF DO NOT CHOOSE PUNCH 1

   1) Cancer
   2) Heart disease
   3) Diabetes
   4) Kidney disease
   5) Lung disease
   6) None of these EXCLUSIVE, DO NOT ROTATE

B. Which of the following types of cancer have you been diagnosed with? Please select all that apply. RANDOMIZE, MULTI RESPONSE, TERMINATE IF DO NOT CHOOSE PUNCH 1

   1) Breast
   2) Colon
   3) Endometrial
   4) Lung
   5) Cervical
   6) Skin
   7) Ovarian
   8) Other DO NOT ROTATE

C. Which of the following breast cancer treatments, if any, have you undergone? Please select all that apply. RANDOMIZE, MULTI RESPONSE, TERMINATE IF DO NOT CHOOSE PUNCH 1 OR 2

1) Mastectomy CODE AS MASTECTOMY
2) Lumpectomy CODE AS LUMPECTOMY
3) Radiation therapy
4) Chemotherapy
5) Hormone therapy
6) Targeted therapy
7) Bone-directed therapy
8) None of these EXCLUSIVE, DO NOT ROTATE

MARITAL. What is your current marital status?

1) Married or living as married CODE AS SIGNIFICANT OTHER
2) In a relationship CODE AS SIGNIFICANT OTHER
3) Single
4) Widowed
5) Separated
6) Divorced

CONTENT QUESTIONS

DISPLAY TO RESPONDENT IF MASTECTOMY: Please consider your **mastectomy** for the purposes of these questions. If you have had more than one, please consider the most recent one.

DISPLAY TO RESPONDENT IF LUMPECTOMY: Please consider your **lumpectomy** for the purposes of these questions. If you have had more than one, please consider the most recent one.

1. Approximately how long was your breast cancer surgery consultation with your surgeon? Please give your answer in minutes. Your best guess is fine. OPEN END NUMERIC, MIN 1, MAX 180

2. When choosing to have breast cancer surgery, which of the following was **more** important to you? RANDOMIZE

    1) Making a quick decision to move the process forward
    2) Thoroughly researching all my options

3. How strongly do you agree or disagree with the following statement – once my breast cancer surgeon recommended a surgery, I did not feel the need to research other options.

    1) Agree strongly
    2) Agree somewhat
    3) Disagree somewhat
    4) Disagree strongly

4. How strongly do you agree or disagree with the following statement – I wish I would have had **more** time to explore different breast cancer surgery options.

    1) Agree strongly
    2) Agree somewhat
    3) Disagree somewhat
    4) Disagree strongly

5. Did you get a second surgical opinion before deciding on a breast cancer surgical treatment? RANDOMIZE

    1) Yes
    2) No

6. How informed or uninformed did you feel about treatment options before undergoing breast cancer surgery?

    1) Completely informed
    2) Somewhat informed
    3) Somewhat uninformed
    4) Completely uninformed

7. Other than your primary breast surgeon, which of the following sources, if any, did you use to research information about breast cancer surgery treatment options? Please select all that apply. RANDOMIZE, MULTI RESPONSE

    1) Books and magazines
    2) Online medical sites, such as WebMD
    3) Online blogs, forums or discussion boards
    4) Physicians other than my primary breast surgeon
    5) Significant other DISPLAY IF SIGNIFICANT OTHER
    6) Family members, other than a significant other

7) Friends
8) In-person support groups
9) Other SPECIFY, DO NOT ROTATE
10) I did not use any sources other than my primary breast surgeon to research breast cancer treatment options EXCLUSIVE, DO NOT ROTATE

DISPLAY TO RESPONDENT IF MASTECTOMY: **Nipple-sparing mastectomy** is a type of breast cancer surgery that allows for complete removal of the breast tissue while keeping the skin, nipple and the areola intact.

8. IF MASTECTOMY Did your physicians tell you about nipple-sparing mastectomy as a potential breast cancer surgery treatment option? RANDOMIZE

   1) Yes
   2) No CODE AS NO NSM

9. IF NO NSM How strongly do you agree or disagree with the following statement – I would have considered nipple-sparing mastectomy if my physicians had told me about it.

   1) Agree strongly
   2) Agree somewhat
   3) Disagree somewhat
   4) Disagree strongly

DISPLAY TO RESPONDENT IF LUMPECTOMY: **Lumpectomies** can be performed using two different types of surgical approaches:

- **Tumor removal only:** an incision is placed directly over or close to the tumor, often leaving a visible scar.
- **Tumor removal plus cosmetic outcome:** an incision is placed further from the tumor, so the scar can be hidden – for example, in the arm pit, the fold underneath the breast or along the areola.

10. IF LUMPECTOMY Did your physician tell you about the option of having an incision in a hidden location, when reviewing your breast cancer surgery treatment options? RANDOMIZE

    1) Yes
    2) No CODE AS NO INCISION OPTION

11. IF NO INCISION OPTION Would you have considered an incision where your scar could be hidden if your physicians had told you about it?

    1) Yes, definitely
    2) Yes, probably
    3) No, probably not
    4) No, definitely not

12. How strongly do you agree or disagree with the following statement – I do not like the location of my surgical scar.

    1) Agree strongly
    2) Agree somewhat
    3) Disagree somewhat
    4) Disagree strongly

13. How often, if ever, do you feel self-conscious due to scars from your breast cancer surgery?

    1) All the time
    2) Some of the time
    3) Rarely
    4) Never

14. How often, if ever, do you decide not to wear a certain piece of clothing because it reveals your breast cancer surgery scars?

    1) All the time
    2) Some of the time
    3) Rarely
    4) Never

15. How strongly do you agree or disagree with the following statement – before my surgery, I did not realize how uncomfortable my breast cancer surgery scars would make me feel when I'm undressed.

    1) Agree strongly
    2) Agree somewhat
    3) Disagree somewhat
    4) Disagree strongly

16. How strongly do you agree or disagree with the following statement – before my surgery, I did not realize how uncomfortable my breast cancer surgery scars would make me feel when **someone else** sees me undressed.

    1) Agree strongly
    2) Agree somewhat
    3) Disagree somewhat
    4) Disagree strongly

DEMOGRAPHIC QUESTIONS

D. Are you covered by any kind of health insurance or health care plan? RANDOMIZE

    1) Yes CODE AS INSURED
    2) No

E. IF INSURED Which kind of health insurance or health care coverage do you have? RANDOMIZE

    1) Medicare
    2) Medicaid
    3) Employment-based
    4) Direct purchase (meaning, you purchased it on your own)
    5) Military healthcare
    6) Other SPECIFY, DO NOT ROTATE
    7) Don’t know/refused DO NOT ROTATE

PARENT. Do you currently have any children under the age of 18 living in your home?

    1) Yes
    2) No

AREA. Do you live in the city, the suburbs, or in a rural area?

    1) City / urban area
    2) Suburbs
    3) Rural area (for example: in a very small town in the country or on a farm)

EDUCATION. What was your last level of formal education?

1) Grade school
2) Some high school
3) Graduated from high school
4) Some college
5) Graduated from college
6) Graduate or post-graduate work
7) Technical or vocational school

EMPLOYMENT. What is your employment status?

1) Work full-time
2) Work part-time
3) Unemployed
4) Retired
5) Stay-at-home / do not work

HISPANIC. These next two questions are just to ensure a proper representation by ethnic groups. Are you of Hispanic, Latino, or Spanish descent?

1) Yes
2) No

RACE. What race or ethnic group are you a member of?

1) Asian
2) African American
3) White or Caucasian
4) Native American
5) Other

INCOME. For statistical purposes only, which of the following categories includes your total household income for last year?

1) Less than $35,000
2) $35,000-$49,999
3) $50,000-$74,999
4) $75,000-$99,999
5) $100,000-$149,999
6) $150,000 or more
