# Supplementary material for: How do breast cancer surgery scars impact survivorship? Findings from a nationwide survey in the United States
Source: BMC Cancer. 2019 Apr 11;19:342. doi: 10.1186/s12885-019-5553-0 (PMC6458748; doi:10.1186/s12885-019-5553-0)
Supplement: Supplementary file 2 — Women Reporting Both Surgeries. This file provides background, results, and commentary on the 132 women who reported both lumpectomy and mastectomy. (PDF 238 kb) [file 12885_2019_5553_MOESM2_ESM.pdf]

How Do Breast Cancer Surgery Scars Impact Survivorship? Findings from a Nationwide Survey in the United States

Jennifer Gass, MD; Sunny Mitchell, MD; Michael Hanna, PhD

Supplemental File 2: Women Who Reported both Lumpectomy and Mastectomy

Background

The survey was completed by 132 women who reported both lumpectomy and mastectomy. The first 100 were assigned to the mastectomy survey and then the rest to the lumpectomy survey. These 132 patients may represent any possible clinical history that would lead a woman to have both surgeries over any time period. It was not originally our intention to analyze their results separately from the women who had either lumpectomy only or mastectomy only, but we eventually realized that we had to remove them into a separate third group, because their results were quite different (both for demographics and survey responses). Due to the variety of possible clinical histories and the assignment to different subsets of study questions and instructions, we found it difficult to interpret the results of this third group on this portion of the survey. Three anonymous peer reviewers also found this group confusing, so we decided to move these results to this supplemental file. We encourage readers to read the main paper in its entirety before attempting to review the results of this third group that reported both surgeries.

Results

The demographic characteristics of this group are presented in the table 1 of this supplemental file.

Supplemental File 2, Table 1. Demographic & Healthcare Characteristics of the Study Subsample of Women Who Reported both Lumpectomy and Mastectomy. In the rows, the demographic characteristics are presented in bold, subtotals of various answer options (if any) are presented in italics, and original answer options are presented in smaller roman type. The results presented are the percentage of the total (n=132). For the sake of reading simplicity, we do not also present the actual number of subjects, but they are available upon request. Due to rounding off, the percentages may not always add up exactly to 100% (or other indicated subtotals).

	**Both Surgeries**
**Age**	
18-29	*9*
30-39	*39*
40-49	*43*
50-59	*3*
60-69	*3*
70-89	*2*
**Race**	
Majority Subtotal	*63*
White or Caucasian	63
Minority Subtotal	*37*
African American	28
Asian	6
Native American	2
Other	1
**Ethnicity**	
Not Hispanic, Latino, or Spanish Descent	85
Hispanic, Latino, or Spanish Descent	15
**Education**	
College Graduate Subtotal	*34*
Graduate or post-graduate work	23
Graduated from college	11
Not College Graduate Subtotal	*66*
Some College	57
Technical or vocational school	0
Graduated from high school	8
Some high school	1
Grade school	0
**Employment**	
Working Full-Time Subtotal	*86*
Work full-time	86
Not Working Full-Time Subtotal	*14*
Work part-time	4
Unemployed	2
Retired	5
Stay-at-home / do not work	4

**Income (total household income for last year)** [a]	
Less than $35,000	7
$35,000 – $49,999	10
$50,000 – $74,999	37
$75,000 – $99,999	17
$100,000 – $149,999	27
$150,000 or more	2
**Residential Area Type**	
City / urban area	36
Suburbs	58
Rural area (e.g. very small town or farm)	5
**Marital Status**	
Has Significant Other Subtotal	*60*
Married or living as married	57
In a relationship	3
Does Not Have Significant Other Subtotal	*40*
Single	20
Separated	1
Divorced	17
Widowed	2
**Parental Status**	
No children under 18 living in her home	35
Has child under 18 living in her home	65
**Covered by Health Insurance or Health Care Plan**	
Yes	88
No	12
**Breast Cancer Treatments Undergone**	
Chemotherapy	26
Radiation therapy	24
Hormone therapy	20
Targeted therapy	20
Bone-directed therapy	15

a) To put the income results into context: the US government reports that the median household income in 2015 was $55,775, and the weighted average poverty threshold was $12,082 for a one-person household, $15,391 for a two-person household, $24,036 for a four-person household in which two were children under 18, and $35,473 for a seven-person household in which five were children under 18.
https://www.census.gov/content/dam/Census/library/publications/2016/demo/acsbr15-02.pdf
http://www.census.gov/data/tables/time-series/demo/income-poverty/historical-poverty-thresholds.html

The scars had a psychosocial impact on nearly all women in the group of patients who reported both surgeries (**figure**, next page). The portion of women who disagreed somewhat or strongly to both questions in the upper row of the figure and also responded “rarely” or “never” to both questions in the lower row of the figure was 3.8%.

Figure. Stacked horizontal bar charts for the results to Q15 (top left), Q16 (top right), Q13 (bottom left), and Q14 (bottom right). Note 1: The bootstrappped 95%CI for the answer "agree strongly" was 15-30% for Q15 and 19-34% for Q16. The bootstrappped 95%CI for the answer "all the time" was 33-49% for Q13 and 19-35% for Q14. Note 2: For Q15 and Q16, 104 women (79%) gave the same answer to both statements; 24 (18%) agreed to both but with different strengths; and 4 (3%) agreed with one statement and disagreed with the other.

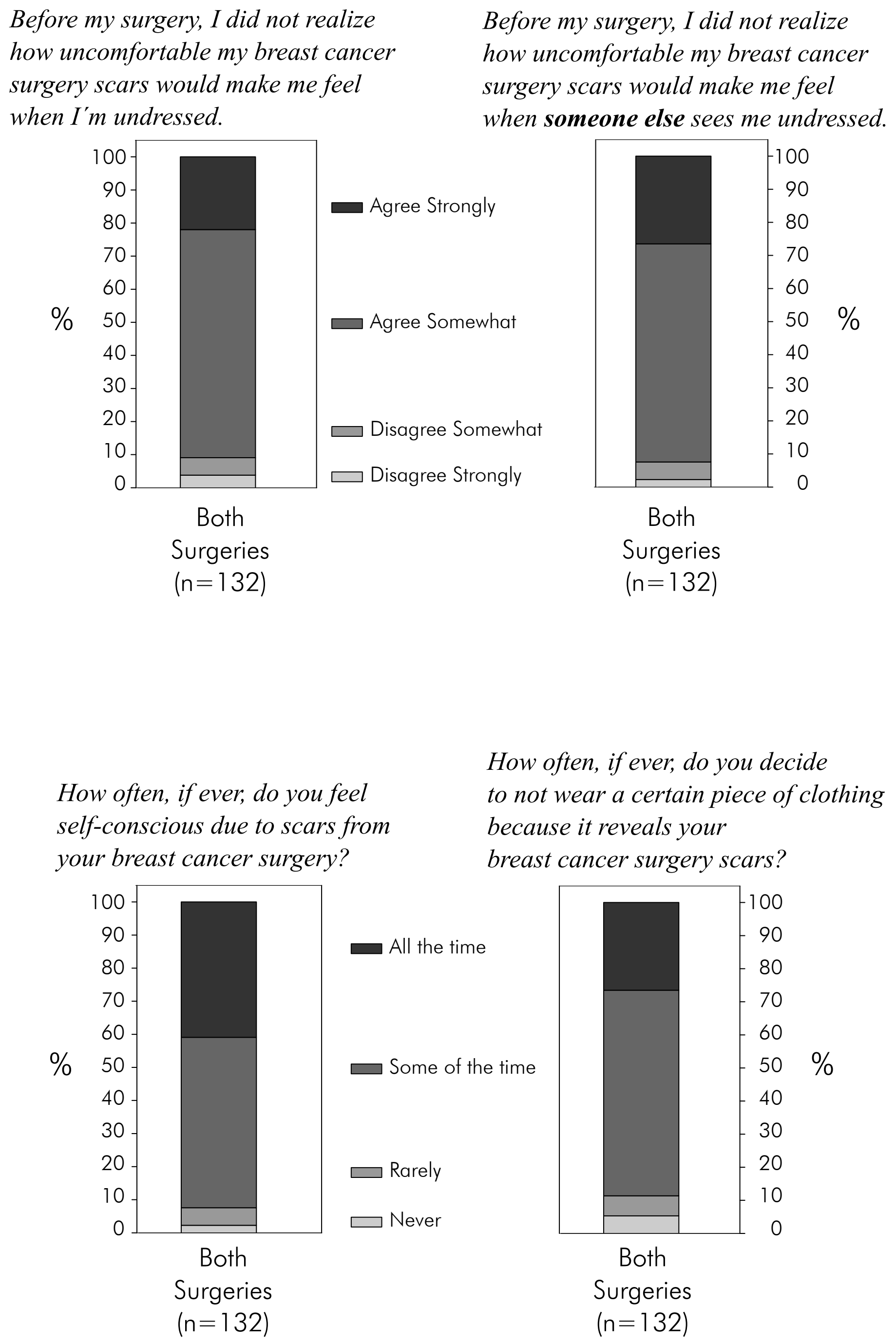

The majority of women agreed that they did not like the location of the scar (**table 2**).

Supplemental File 2, Table 2. Descriptive Results for Agreement / Disagreement with the Main Outcome, "I do not like the location of my surgical scar (Q12).

	n	%	95% CI
**Both Surgeries (n=132)**			
Agree strongly	31	24	17 - 31
Agree somewhat	89	67	58 - 75
Disagree somewhat	10	8	4 - 13
Disagree strongly	2	2	0 - 4

Regression analysis suggested that women who were college graduates had substantially higher odds of strong agreement that they did not like the location of the scar (table 3).

Supplemental File, Table 3. Regression analysis for strong agreement with the statement, "I do not like the location of my surgical scar" (Q12).

	OR	lower 95%CI	upper 95%CI	p
**Both Surgeries (n=132)**				
College Graduate	15	5.0	44	<0.001
Significant Other	0.6	0.2	1.9	0.4
Age (years)	0.99	0.94	1.03	0.5

In the both-surgeries group, 83% reported that they were told about nipple-sparing mastectomy or hidden-incision lumpectomy (depending on their survey assignment). The sample size of the remaining subgroups who had not heard about these options (n=5 & n=17) are far too small for their results on Qs 9 & 11 to be reliable (details not shown), but their response rates were quite similar to those for the other groups from the corresponding survey shown on the right side of figure 2 in the main paper.

Commentary

Although some of these patients were instructed to answer the questions with reference to their most recent lumpectomy, such a distinction was probably difficult to make in regards to the questions about surgical scarring reported here. Thus their answers are probably often equivalent to the rest of the both-surgeries group who were instructed to answer in regards to their most recent mastectomy. The results among this both-surgeries group were noticeably worse than in the groups that reported lumpectomy only or mastectomy only. This may in part reflect the fact that the group reporting both surgeries was also quite different demographically, and it may in part reflect more extensive scarring. Due to the variety of possible clinical histories that would lead to having both surgeries, it is difficult to interpret the exact meaning of the results in this group, but it is in any case clear that the scars bothered them as much or more than women who had only one form of surgery.
